# Identification of N6-Methyladenosine-Related Factors and the Prediction of the Regulatory Mechanism of Hair Follicle Development in Rex and Hycole Rabbits

**DOI:** 10.3390/biology12111448

**Published:** 2023-11-17

**Authors:** Gang Luo, Ruiguang Gong, Yaotian Ai, Tongyan Zhu, Zhanjun Ren

**Affiliations:** 1 College of Animal Science and Technology, Northwest A&F University, Xianyang 712100, China; luogang66@nwafu.edu.cn (G.L.); gongwrong@nwafu.edu.cn (R.G.); 18581899494@163.com (Y.A.); zhutongyan234@nwafu.edu.cn (T.Z.); 2 College of Animal Science, Fujian Agriculture and Forestry University, Fuzhou 350000, China

**Keywords:** rex rabbit, Hycole rabbit, hair, hair follicle, m^6^A

## Abstract

**Simple Summary:**

N6-methyladenosine (m^6^A) is an important modification for genes. Hair follicle development is crucial for the animal fur economy. To improve the quality of animal fur and solve the problem of baldness in people, we explored the regulatory mechanism of m^6^A on rabbit hair follicles and found that five methylases regulated the development of hair follicles through differential genes/signal pathways. These findings laid a molecular foundation for improving the quality of animal fur and solving the problem of baldness in people.

**Abstract:**

Hair follicle development directly affects the development of the rabbit fur industry. The growth and development of a hair follicle is modified and regulated by many genes and mechanisms. M^6^A is an important RNA modification. However, there are few studies on the effects of the regulation of m^6^A on hair follicle growth and development. In this study, hematoxylin–eosin (HE) staining was used to explore the difference in hair follicle development between Rex rabbits and Hycole rabbits, and we performed m^6^A sequencing to identify the key genes with m^6^A modification in hair follicle growth. The results showed that the hair length, coarse hair percentage, primary hair follicle ratio, and skin thickness of Hycole rabbits were significantly higher than those of Rex rabbits. However, the proportion of secondary hair follicles in Hycole rabbits was significantly lower than that in Rex rabbits. In addition, we found five differential methylases, 20 differential genes, and 24 differential signaling pathways related to hair growth and development. The results of the Sankey diagram showed that 12 genes were related to 13 signal pathways. Finally, we found that five methylases regulated the development of hair follicles through differential genes/signal pathways. These findings laid a molecular foundation for the function of m^6^A modification in hair development.

## 1. Introduction

Rex rabbits are a type of rabbit with high hair density, capillary, and short hair. Hycole rabbits are a type of rabbit with low hair density and thick and long hair. The hair quality of rabbits is closely related to the hair density, which is mainly determined by the hair follicle density [1]. Therefore, the study of hair follicle development is of great significance to rabbit fur production. Hair follicle cycling is a complex biological process [2]. Hair follicles undergo anagen, catagen, and telogen cycles [3]. Many mRNAs and miRNAs are involved in the formation of hair follicles [4,5,6]. Hair follicle morphogenesis depends on many signaling pathways such as WNT, Shh, p53, TGF-β, Notch, and BMP [7]. However, the regulation pathways and methods of m^6^A modification on hair-follicle-development-related mRNAs in rabbits is unknown.

M^6^A is a common RNA modification [8]. At present, it has been found that m^6^A modification has been carried out under the action of methylase (METTL5, METTL14, METTL3, WTAP, and METTL4) and demethylase (FTO and ALKBH5) [9,10,11,12,13]. In addition, “reader” proteins containing YTHDC1, IGFBP2 and other YTH domain proteins are also determining factors in the m^6^A modification process [13,14,15,16,17,18]. These enzymes play an important role in animal growth [19], fat metabolism [20], reproduction [21], and other physiological processes.

In this study, we identified methylases, methylated genes, and signal pathways in Rex rabbit and Hycole rabbit skin by the MeRIP-seq method. Based on existing studies, we found five different methyltransferases, 20 methylation-modified differential genes, and 24 differential signaling pathways related to hair follicle development in Rex rabbits and Hycole rabbits. Finally, we found that 13 signal pathways were regulated by 12 genes among the genes and signal pathways we selected. In addition, we found that five methylases mediated 20 methylated genes to regulate hair follicle development through multiple pathways based on existing studies. This study lays a molecular theoretical foundation for further exploring the regulation of rabbit hair follicle development by m^6^A modification.

## 2. Material and Methods

### 2.1. Animals

Three newborn female Rex rabbits and three newborn female Hycole rabbits were used for methylated RNA immunoprecipitation sequencing (MeRIP-seq) [22]. Three 165-day-old Rex rabbits and three 165-day-old Hycole rabbits were used for photographing, hair index determination, and HE staining. All rabbits were collected from the same farm of the Northwest A&F University (Yangling, Shannxi, China). The rabbit farm belongs to Professor Ren Zhanjun, and he permitted the experiment.

### 2.2. Hair Index Determination

Pictures of Rex rabbits and Hycole rabbits were taken with a high pixel mobile phone (64 million pixels). The calculation method for the proportion of primary hair follicles was as follows: Firstly, we selected three 0.575 × 0.862 m^2^ microscope fields. Then, we recorded the number of hair follicles and obtained the number of primary hair follicles and all hair follicles per unit area. Finally, the proportion of primary hair follicles was calculated. We collected approximately 0.003 g of rabbit hair and recorded its weight as T1 (*n* = 3). The coarse wool was selected, and we recorded its weight as T2. Coarse wool ratio = T2/T1 × 100%. The hair length was directly measured by vernier caliper after collection.

### 2.3. Hematoxylin–Eosin (HE) Staining

A proper amount of rabbit back skin tissue was collected and fixed in formaldehyde solution. Then, the skin tissues were treated, embedded, sectioned and stained. The specific method is the same as that in the previous study [23]. Briefly, alcohol was used to remove the water in the tissue block, and then xylene was used to replace the alcohol in the tissue block. Tissue blocks were embedded in paraffin and cut into thin sections on a microtome. The slices were sequentially placed in xylene I (8 min), xylene II (8 min), anhydrous ethanol I (6 min), anhydrous ethanol II (6 min), 95% alcohol (6 min), 85% alcohol (6 min), and 75% alcohol (5 min) and rinsed with running water. Slices were stained with Harris hematoxylin for 3–8 min and rinsed with tap water. Then, the slices were differentiated with 1% hydrochloric acid alcohol for a few seconds and rinsed with tap water. The eosin staining solution was used to stain the slices for 1–3 min. Subsequently, we sequentially placed the slices in 75% alcohol (30 s), 85% alcohol (30 s), 95% alcohol I (1 min), 95% alcohol II (2 min), anhydrous ethanol I (5 min), anhydrous ethanol II (5 min), xylene I (5 min), and xylene II (7 min) to dehydrate and become transparent. Finally, we took the slices out of xylene and let them dry slightly; then, we sealed them with neutral gum. The primary hair follicle ratio and skin thickness were measured for each sample with a light microscope.

### 2.4. RNA Fragmentation

RNA was extracted from skin of rabbits by TRIzol reagent (Invitrogen Co., Carlsbad, CA, USA). Agilent 2100 Bioanalyzer (Agilent, Santa Clara, CA, USA) was used to assess the concentration and integrity of the RNA. The poly (A) RNA was fragmented into small pieces using Magnesium RNA Fragmentation Module (NEB, cat.e6150, Ipswich, MA, USA) under 86 °C, 7 min. Fragmentation buffer was used to break the mRNA into ∼100 nucleotides fragments.

### 2.5. M^6^A IP and Library Construction

The fragmented mRNA were divided into immunoprecipitation (IP) (95%) and IP control (input) groups (5%). We followed the instructions of the m^6^A RNA methylation library construction kit (A&D technology, Beijing, China) to IP RNA. Briefly, the cleaved RNA fragments were incubated for 2 h at 4 °C with m^6^A-specific antibody (No. 202003, Synaptic Systems, Göttingen, Germany) in IP buffer (50 mM Tris-HCl, 750 mM NaCl, and 0.5% Igepal CA-630). Then the IP RNA was reverse transcribed by SuperScript™ II Reverse Transcriptase (Invitrogen, cat. 1896649, Carlsbad, CA, USA). After, we performed the heat-labile UDG enzyme (NEB, cat.m0280, Ipswich, MA, USA) treatment of the U-labeled second-stranded DNAs. Then, the mRNA reacted with the antibody (binding to m^6^A modification site) with magnetic beads, and sequencing with high-throughput (Illumina Novaseq™6000) (LC-Bio Technology Co., Ltd., Hangzhou, China, 2020) was performed. 

### 2.6. RT-qPCR

The Prime Script RT Reagent Kit (Takara Bio, Saint-Germain-en Laye, France) was used to reverse transcribe the total RNA. RT-qPCR experiments were carried out with a 10 µL system by SYBR Green. β-Actin was used as an internal control. All the primers used for qPCR are listed in Table 1.

### 2.7. Data Analysis

After sequencing, we used fastp (https://github.com/OpenGene/fastp, accessed on 29 May 2022) [24], the comparison tool bowtie2 [25], and HISAT2 (2.2.1.0) (http://daehwankimlab.github.io/hisat2, accessed on 24 July 2020) [26] software to filter, remove, and compare raw reads separately. The R-Pack exomepeak2 (https://bioconductor.org/packages/edgeR, accessed on 19 May 2022) [27] and DiffBind (3.5) [28] software were used to merge peaks between groups and calculate the abundance of peaks in each sample. StringTie (2.2.1) (https://ccb.jhu.edu/software/stringtie) was used to perform the expression level on 20 May 2022 for all mRNAs from Input libraries by calculating the FPKM (total exon fragments /mapped reads (millions) × exon length (kB)). The differentially expressed mRNAs were selected with log2 (fold change) > 1 or log2 (fold change) < −1 and *p* value < 0.05 by R package edgeR (4.0.1) (https://bioconductor.org/packages/edgeR on 20 May 2022). Genomes (KEGG) pathway analysis was performed using the database for annotation, visualization, and integrated discovery [29]. Hair and follicle data results were presented as the mean ± standard deviation (SD). GraphPad Prism7 (GraphPad Software, La Jolla, CA, USA) was used to assess the difference. The Student’s *t*-test was used to analyze the significance of the different levels.

## 3. Results

### 3.1. Difference in Hair Follicles between Rex and Hycole Rabbits

**Figure 1:**
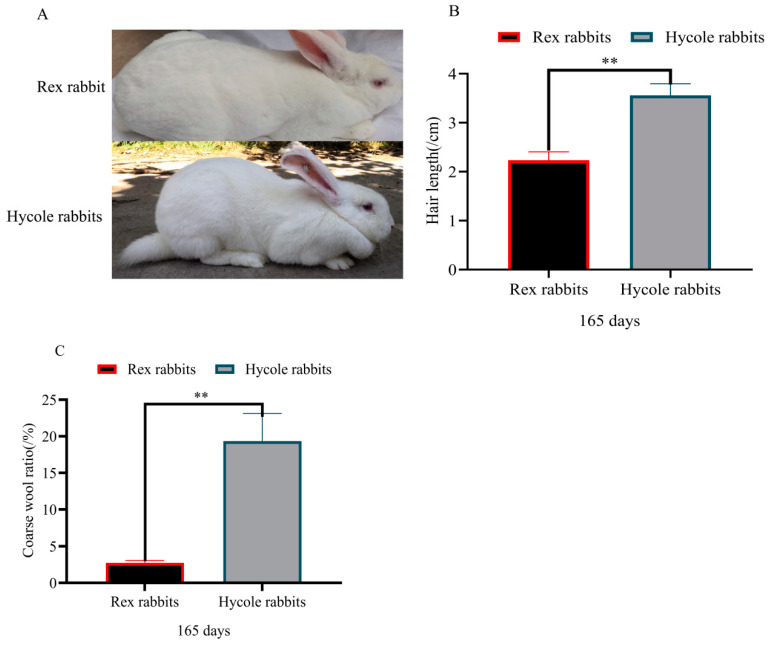
Hair difference between Rex Rabbit and Hycole rabbit. (**A**) Pictures of Rex rabbits and Hycole rabbits; (**B**) hair length of Rex rabbits and Hycole rabbits; (**C**) coarse wool ratio of Rex rabbits and Hycole rabbits (“**”, *p* ≤ 0.01).

**Figure 2:**
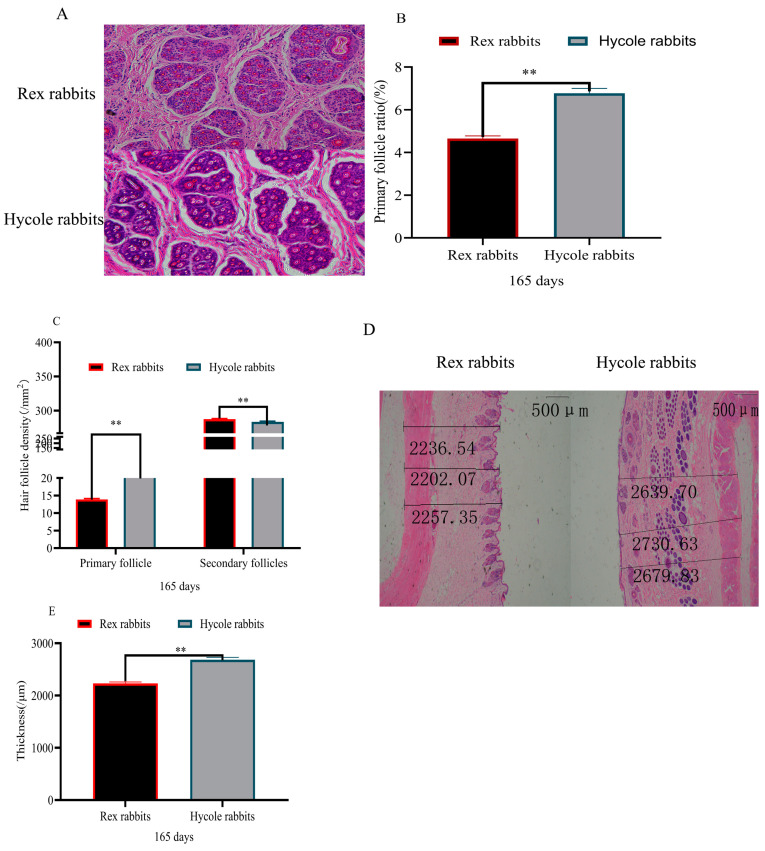
Hair follicle distribution and skin thickness. (**A**) HE staining cross section of skin; (**B**) proportion of primary hair follicles; (**C**) primary and secondary follicle density; (**D**) HE staining longitudinal section of skin; (**E**) skin thickness (longitudinal section of skin in Rex rabbits and Hycole rabbits) (“**”, *p* ≤ 0.01).

Based on the pictures of the Rex rabbits and Hycole rabbits, we found that the fur of the Rex rabbits was different from that of Hycole rabbits (Figure 1A). The results showed that the hair of the Hycole rabbits was significantly longer than that of Rex rabbits (*p* < 0.01) (Figure 1B). In addition. the coarse hair rate of the Hycole rabbits was also significantly higher than those of the Rex rabbits (*p* < 0.01) (Figure 1C). The proportion of primary hair follicles of the Rex rabbits was significantly lower than that of the Hycole rabbits (*p* < 0.01) (Figure 2A,B). At the same time, the primary hair follicle ratio of Hycole rabbits was significantly higher than that of the Rex rabbits, whereas the ratio of the secondary hair follicles of the Rex rabbits was significantly higher than that of the Hycole rabbits (*p* < 0.01) (Figure 2C). The results showed that the skin of the Rex rabbit was significantly thinner than that of the Hycole rabbit (*p* < 0.01) (Figure 2D,E).

### 3.2. Summary and Quality Control of Rabbit m^6^A Sequencing Data

As shown in Supplementary Table S1, MeRIP-seq produced 65,627,376–97,648,830 raw reads from input or IP skin tissues from Rex rabbit (ski) and Hycole rabbit (FYM). We found the GC content in the Rex rabbits’ IP and input was lower than that in the Hycole rabbits. In addition, the proportion of unique mapped reads was higher than 62.30%, and the proportion of multi-mapped reads varied from 3.27% to 25.64% (Supplementary Table S2). 

### 3.3. General Features of Rabbit m^6^A Methylation

**Figure 3:**
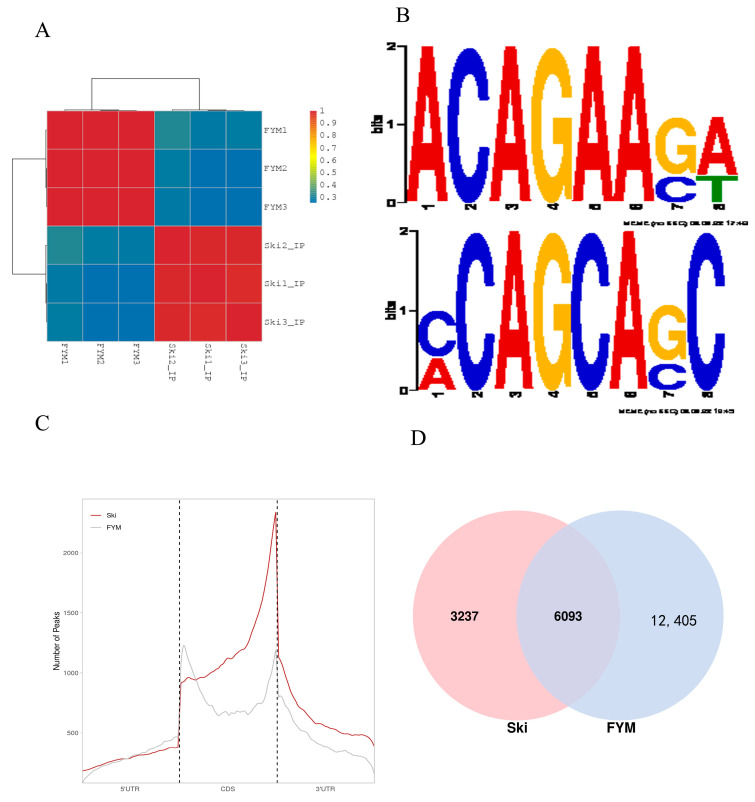
(**A**) Cluster analysis heat map; (**B**) sequence logo identified from Rex rabbit and Hycole rabbit skin; (**C**) overlap of m^6^A peaks from Rex rabbit and Hycole rabbit skin; (**D**) distribution of m^6^A peaks across the length of mRNA.

The samples were clustered by calculating the correlation coefficient between the Rex rabbit skin samples and the Hycole rabbit skin samples, which indicated good uniformity within the group (Figure 3A). As shown in Figure 3B, m^6^A-modified classical sequences RRACH appeared in the sequencing results of the Rex rabbits and Hycole rabbits. According to the statistics, 6093 peaks were methylated both in the Rex rabbit skin and the Hycole rabbit skin. Further, 3237 and 12,405 peaks were specifically methylated in the Rex rabbits’ skin and the Hycole rabbits’ skin, respectively (Figure 3C). To explore the preferential localization of m^6^A, we counted the distribution of peaks and found that in the CDS, the start and stop codons were the main areas of m^6^A (Figure 3D). In addition, the peaks enriched in the Rex rabbits were higher than those in the Hycole rabbits in the start codons (Figure 3D). However, the peaks enriched in the Rex rabbits were lower than those in Hycole rabbits in the stop codons (Figure 3D). 

### 3.4. KEGG Pathway, Methylases, and Methylation Modifying Genes in Rex Rabbit Skin and Hycole Rabbit Skin

**Figure 4:**
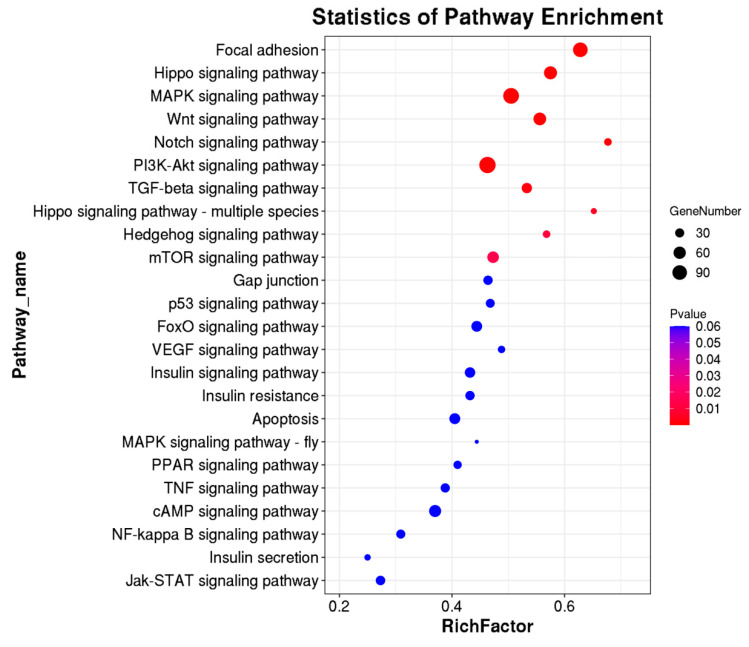
Enrichment pathway of the m^6^A peak related to hair growth and development.

**Figure 5:**
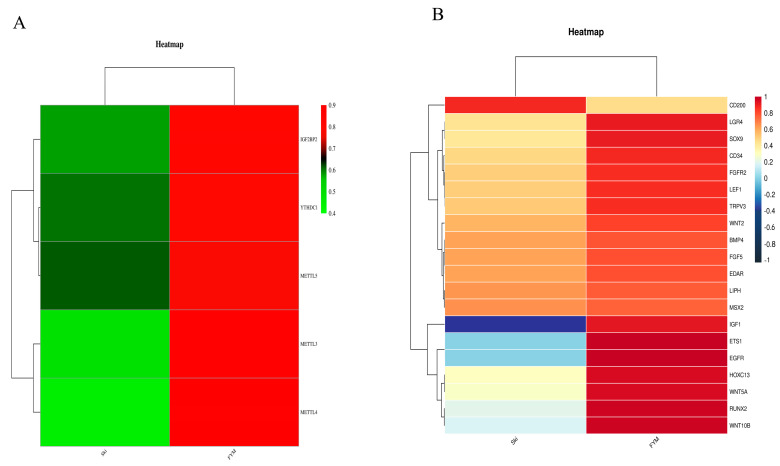
(**A**) Cluster heat map of differential methylases from Rex rabbit and Hycole rabbit skin; (**B**) cluster heat map of differential genes related to hair development from Rex rabbit and Hycole rabbit skin.

**Figure 6:**
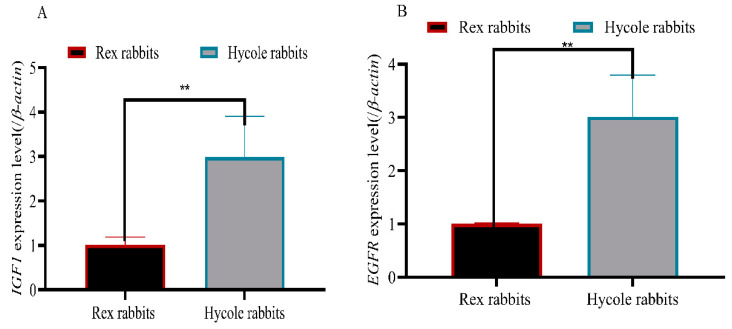
(**A**) *IGF1* expression level in Rex rabbit and Hycole rabbit skin; (**B**) *EGFR* expression level in Rex rabbit and Hycole rabbit skin; (“**”, *p* ≤ 0.01).

**Figure 7:**
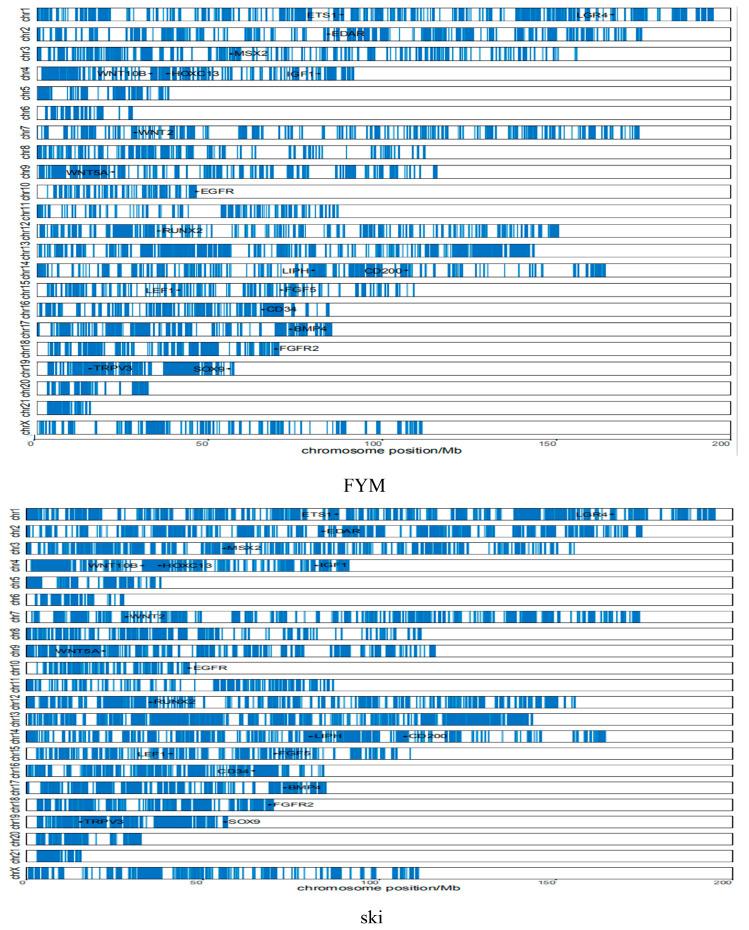
Distribution of differential genes on the chromosomes of 20 genes’ m^6^A modification.

To predict the m^6^A-modified functions associated with hair follicle development, we analyzed the KEGG pathway and found there were 24 differential pathways involved in hair follicle development including Focal adhesion, Hippo, MAPK, WNT, cAMP, and other signaling pathways containing multiple genes (Figure 4). In addition, we found five differential methylation enzymes and 20 differential methylation modification genes were involved in hair follicle development based on the existing study results (Figure 5). To verify the results, we randomly selected two genes (IGF1 and EGFR) for RT-qPCR validation. The expression levels of the *IGF1* gene and EGFR gene in the Rex rabbit skin were significantly higher than those in the Hycole rabbit skin (*p* < 0.01) (Figure 6). In addition, we found the distribution of the m^6^A modification of 20 genes on chromosomes was significantly different (Figure 7).

### 3.5. Regulation of Methylation Modified Genes by Methylase

**Figure 8:**
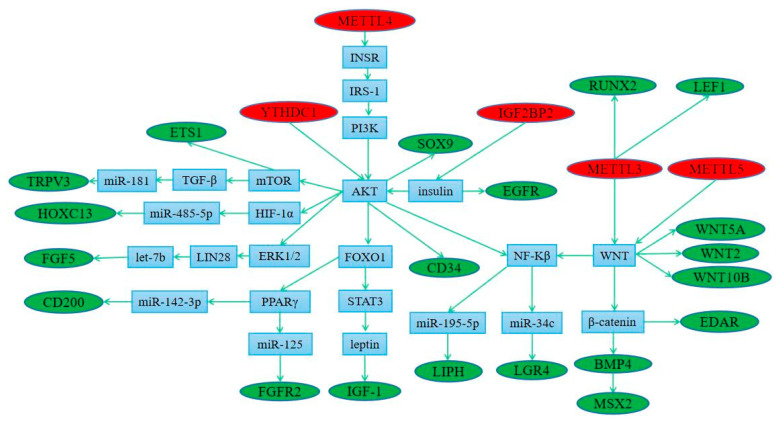
Pathway map of key genes regulated by five methylases.

As shown in Supplementary Figure S1, we found there were many m^6^A modification sites in 20 different genes. According to the existing research, *YTHDC1* can directly regulate the expression of *AKT* (Figure 8). At the same time, *METTL4* and *IGF2BP2* can regulate *AKT* expression through *PI3K* and insulin, respectively. *AKT* can directly regulate the expression of *CD34* and *SOX9* and indirectly regulate the expression of the *ETS1*, *TRPV3*, *HOXC13*, *FGF3*, *CD200*, *FGFR2*, *IGF1*, *LIPH,* and *LGR4* genes (Figure 8). Another regulatory pathway shows that *IGF2BP2* can regulate the expression of the *EGFR* gene through insulin (Figure 8). As shown in Figure 8, *METTL3* can indirectly regulate the expression of the *RUNX2* and *LEF1* genes. At the same time, *METTL3* and METTL5 can regulate the expression of the *WNT2*, *WNT10B*, *WNT5A*, *EDAR*, *BMP4*, *MSX2*, *LIPH,* and *LGR4* genes (Figure 8).

### 3.6. The Connection between Differential Pathways and Differential Genes

**Figure 9:**
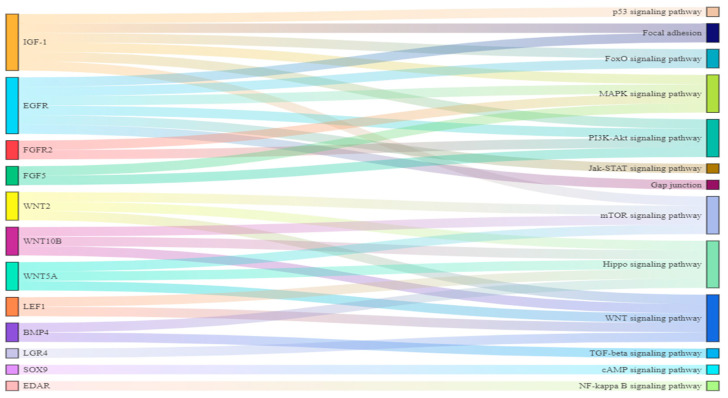
Sankey diagram of genes and signal pathways.

By drawing a Sankey diagram, we found *SOX9*, *LGR4,* and *EDAR* were important regulators in the *cAMP*, *WNT,* and *NF-Kβ* signaling pathways, respectively. *BMP4*, *LEF1*, *FGF2,* and *FGFR2* were involved in the regulation of the two signaling pathways. In addition, *WNT2*, *WNT10B,* and *WNT5A* regulated three signal pathways. *IGF1* and *EGFR* regulated six signal pathways (Figure 9). So, We selected *IGF1* and *EGFR*, which had the most regulated signaling pathways, for PCR validation.

## 4. Discussion

A hair follicle is an important skin derivate with a unique structure and periodic regeneration ability, which plays an important role in hair growth [30]. A hair follicle mainly regulates the growth, color, and fixation of hair [2]. The development of hair follicles plays an important role in the fur rabbit industry [31]. We found that the fur of Rex rabbits was shorter and denser than that of Hycole rabbits. Studies have shown that single nucleotide deletion in exon 9 (1362delA) of LIPH is the reason for the hair phenotype of Rex rabbits [32]. The average hair length, the proportion of primary hair follicles, and the thickness of skin in Hycole rabbits were significantly higher than those in Rex rabbits. The diameter of the primary hair follicles and the volume of their papillae were significantly larger than those of he secondary hair follicles. However, the differentiation and proliferation of secondary hair follicles were faster, which is beneficial for temperature regulation. Therefore, Rex rabbits and Hycole rabbits are two ideal experimental animals to study the differences in hair volume, hair length, and hair follicle growth.

In order to study the mechanism of m^6^A modification on hair growth and development, we selected the skin tissues of Rex rabbits and Hycole rabbits for MeRIP-seq and found many differences in gene modification. Firstly, we found the typical m^6^A motif RRACH of animals and plants in two kinds of rabbits [33,34], and the motifs of the two kinds of rabbits are different at many gene loci in Figure 3B. We also found that the location and distribution of the peaks were significantly different in the two breeds of rabbits. Previous studies have shown that m^6^A methylation regulated the expression of genes related to cashmere growth [35]. These results suggested that m^6^A regulates hair development.

Based on the existing study results, we found that five methylases can regulate different genes through multiple pathways. *METTL3* can directly regulate the expression of *RUNX2* and *LEF1* [36,37]. In addition, *METTL3* and *METTL5* both regulated the *WNT* signaling pathway [38,39]. *WNT2*, *WNT5A,* and *WNT10B* are key factors in the *WNT* signal pathway. Wnt/β-catenin acts on the upstream of *BMP4* [40] and regulates *EDAR* and *MSX2* through β-catenin and *BMP4*, respectively [41,42]. At the same time, *MAPK* regulates chondrocytes apoptosis through *WNT*/*NF-κB* pathways [43]. *NF-κB* plays a role in the expression of *LGR4* and *LIPH* through microR-34c and miR-195-5p, respectively [44,45,46,47]. Knockdown of *METTL4* led to downregulation and inactivation of the *INSR* pathway [48], thereby regulating the *IRS-1*/*PI3K*/*AKT* pathway to improve insulin resistance [49]. In addition, *AKT* regulates the expression of the *ETSI* [50], *TRPV3* [51,52], *HOXC13* [53,54,55], and *FGF5* [56,57,58] genes through the *mTOR*, *mTOR*/*TGF-β*/miR-181, *HIF-1a*/miR-485-5p, and *ERK_1/2_*/*LIN28*/*let-7b* signaling pathways, respectively.

The functions of *IGF2BP2* are associated with insulin resistance [59,60], and insulin regulates the *EGFR* gene to promote the migration of human corneal epithelial cells [61]. In addition, insulin can inhibit hepatic gluconeogenesis by activating the *AKT*/*FOXO1* signaling pathway [62], and *FOXO1* inhibits leptin regulation by blocking *STAT3* interaction [63]. Leptin and estradiol interact to regulate the expression of *IGF-1* [64]. In addition, *FOXO1* transrepresses *PPARγ* transactivation to regulate miR-142-3p, and *CD200* is a target gene of miR-142-3p [65,66,67]. At the same time, *PPARγ* regulates keratinocyte proliferation by targeting *FGFR2* with miR-125b [68,69]. MiR-451a represses the *AKT*/*mTOR* signaling pathway [70], and *AKT* regulates *SOX9* expression [71] to maintain the imatinib-resistant phenotype of CML CD34+ cells [72]. These existing studies have shown that five methylases can regulate the expression of 20 methylation-modified genes. However, the specific regulation mode is still unclear. This study provides a new possibility for further study on how the five methylases regulate 20 methylated-modified genes.

To further explore the regulation of methylation-modified genes on hair follicle growth and development, we associated methylation-modified genes with differential signaling pathways through a Sankey diagram. As shown in Figure 8, there were 12 genes that regulated 13 differential signaling pathways through different pathways. At the same time, we found that 13 signaling pathways played an important role in the growth and development of hair follicles and hairs. *NF-kappaβ* promotes hair follicle growth [73]. The *FOXO* signaling pathway mediates the changes in epidermal morphology, which is closely related to the development of hair follicles [74]. Focal Adhesion Signaling plays an important role in Cochlear Hair Cell Morphology [75]. Some canonical pathways such as *WNT*, *MAPK*, *TGF-β,* and Hippo signaling pathways were detected as promoting the hair follicle growth [76,77,78]. The *PI3K*/*AKT* [79], *mTOR* [79], *JAK*-*STAT* [80], Gap junction [81], and *cAMP* [82] signaling pathways are involved in the development of hair follicles and hairs. In addition, *p53* regulated hair follicle regression [83]. So, 12 methylation modification genes regulate the development of hair follicles and hairs through 13 differential signaling pathways.

## 5. Conclusions

In summary, this study analyzed the modification of m^6^A methylation in Rex and Hycole rabbit skin tissue. We found five differential methylases, 20 differential genes, and 24 differential signaling pathways related to hair development in Rex and Hycole rabbits. Five methylases regulated the expression of 20 genes related to hair follicle development, of which 12 genes were found to regulate 13 important hair follicle signaling pathways. The development of hair follicles directly affected the growth and density of hair. Studying the effect of m^6^A on the development of hair follicles lays a theoretical foundation for m^6^A modification to regulate the development of rabbit hair follicles.

## Figures and Tables

**Table 1:** Primers used in this study.

Gene Name	Primer Sequence (5′-3′)	Tm (°C)
*β* *-actin*	GGAGATCGTGCGGGACAT	60
	GTTGAAGGTGGTCTCGTGGAT	
*IGF1*	ACCCACCCTAACCTGCTGTA	60
	TCCTGTGGGCTTGTTGAAAT	
*EGFR*	ACCTTGTCATTCAGGGGGATG	60
	ACACAAGCCATGGTGGAACT	

## Data Availability

Raw data have been uploaded to NCBI, which are deposited under SRA BioProject accession PRJNA863730.

## Supplementary Materials

The following supporting information can be downloaded at: https://www.mdpi.com/article/10.3390/biology12111448/s1, Figure S1: The abundance of m^6^A in the Rex rabbit and Hycole rabbit skin; Table S1: Summary of reads quality control; Table S2: Summary of reads mapping to the rabbit reference genome.
