# Supplementary material for: Identification of N6-Methyladenosine-Related Factors and the Prediction of the Regulatory Mechanism of Hair Follicle Development in Rex and Hycole Rabbits
Source: Biology (Basel). 2023 Nov 17;12(11):1448. doi: 10.3390/biology12111448 (PMC10669094; doi:10.3390/biology12111448)
Supplement: Supplementary file 1 [file biology-12-01448-s001.zip › biology-2614158-supplementary.pdf]

**Supplement Table S1. Summary of reads quality control.**

| Sample | Raw_Reads | Q20% | Q30% | GC% |
|---|---|---|---|---|
| Ski1-IP | 86509634 | 97.95 | 94.03 | 51.39 |
| Ski2-IP | 71769462 | 97.96 | 94.06 | 52.10 |
| Ski3-IP | 97648830 | 98.10 | 94.32 | 51.16 |
| FYM1-IP | 95927200 | 98.04 | 94.86 | 54.02 |
| FYM2-IP | 85645472 | 98.27 | 95.25 | 54.14 |
| FYM3-IP | 90783892 | 98.20 | 95.12 | 54.03 |
| Ski1-input | 80427926 | 97.95 | 93.96 | 50.25 |
| Ski2-input | 95144464 | 97.87 | 93.80 | 50.85 |
| Ski3-input | 76040226 | 98.04 | 94.10 | 50.62 |
| FYM1-input | 65633142 | 97.84 | 93.63 | 53.23 |
| FYM2-input | 65627376 | 97.67 | 93.33 | 53.24 |
| FYM3-input | 87067342 | 97.94 | 93.96 | 54.11 |

**Supplement Table S2. Summary of reads mapping to the rabbit reference genome**

| Sample | Valid reads | Mapped reads | Unique mapped reads | Multi mapped reads |
|---|---|---|---|---|
| Ski1-IP | 84433594 | 77035455(91.24%) | 57799897(68.46%) | 19235558(22.78%) |
| Ski2-IP | 69573620 | 63058511(90.64%) | 48206148(69.29%) | 14852363(21.35%) |
| Ski3-IP | 95145912 | 87122746(91.57%) | 66412571(69.80%) | 20710175(21.77%) |
| FYM1-IP | 44435680 | 29582015(66.57%) | 27843777(62.66%) | 1738238(3.91%) |
| FYM2-IP | 40231350 | 26614523(66.15%) | 25121280(62.44%) | 1493243(3.71%) |
| FYM3-IP | 41674194 | 27607810(66.25%) | 25964269(62.30%) | 1643541(3.94%) |
| Ski1-input | 78927016 | 73396127(92.99%) | 53160162(67.35%) | 20235965(25.64%) |
| Ski2-input | 93057700 | 86119450(92.54%) | 62593892(67.26%) | 23525558(25.28%) |
| Ski3-input | 74385726 | 69170798(92.99%) | 50984231(68.54%) | 18186567(24.45%) |
| FYM1-input | 65037230 | 49538616(76.17%) | 47339413(72.79%) | 2199203(3.38%) |
| FYM2-input | 64467766 | 48916621(75.88%) | 46809471(72.61%) | 2107150(3.27%) |
| FYM3-input | 86073096 | 64785290(75.27%) | 61697031(71.68%) | 3088259(3.59%) |

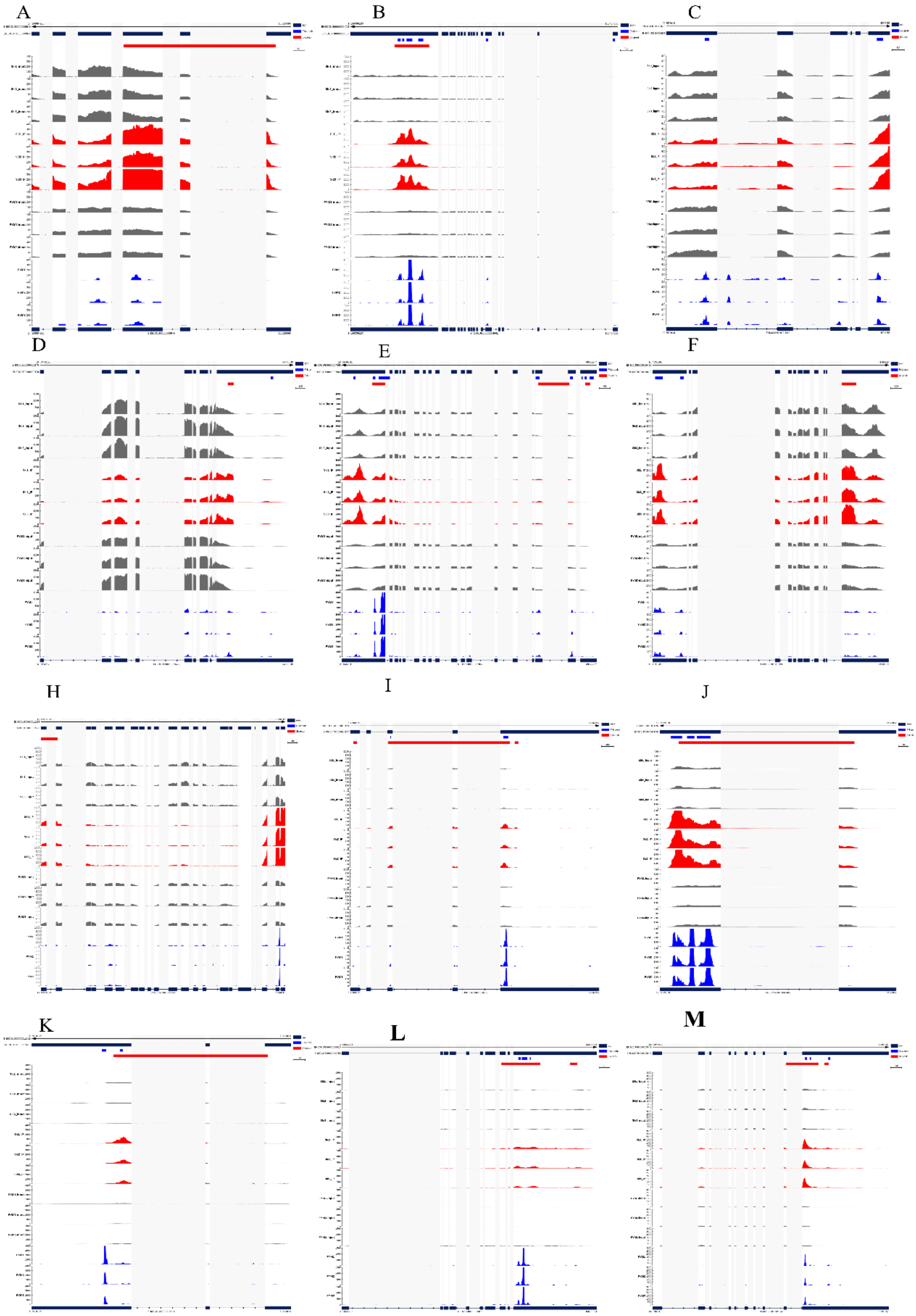
A
B
C
D
E
F
H
I
J
K
L
M

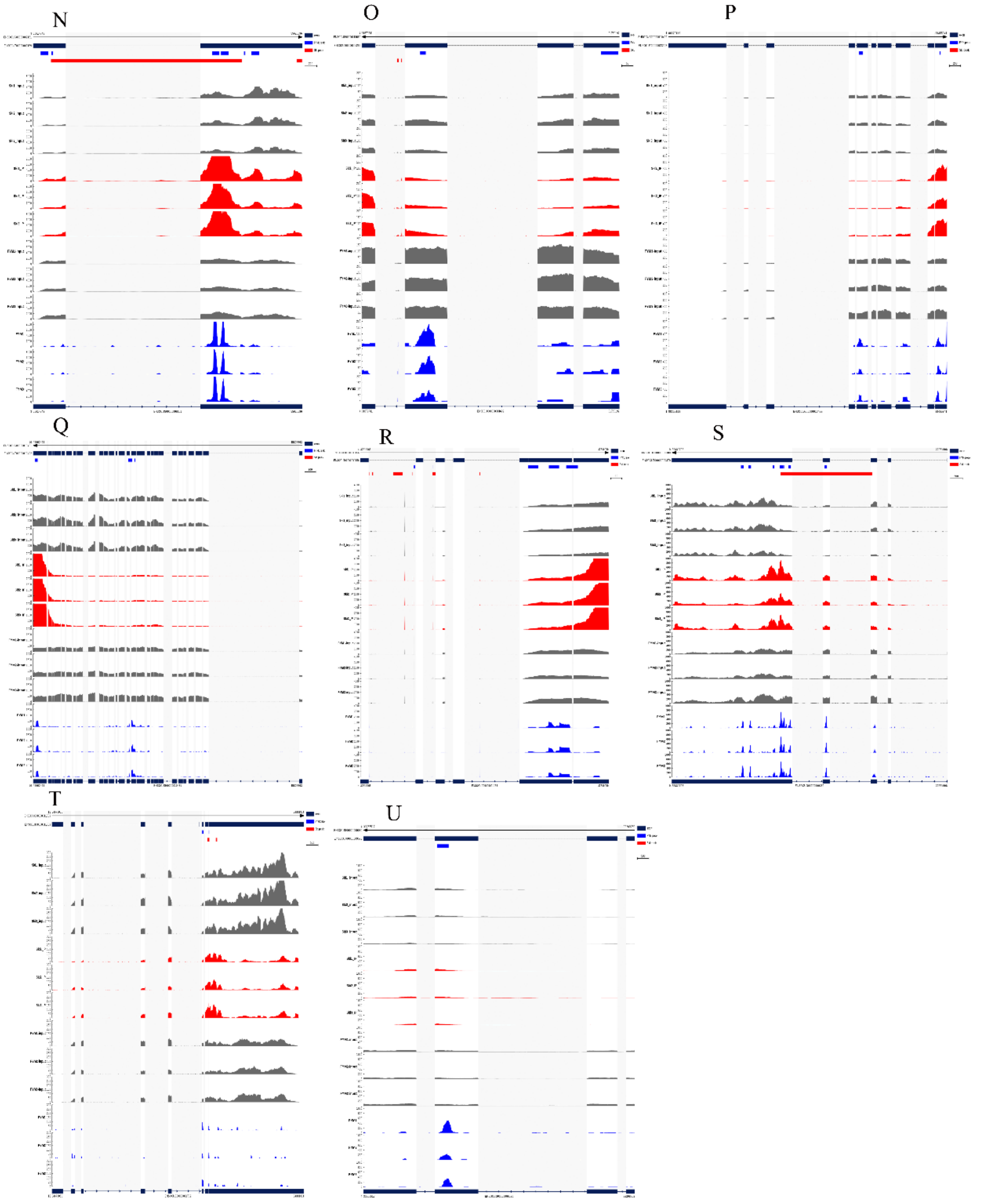


**Supplement Figure S1.The abundance of m$^6$A in the Rex rabbit and Hycole rabbit skin** (A) M$^6$A methylated peaks of CD200 mRNA; (B) M$^6$A methylated peaks of LGR4 mRNA; (C) M$^6$A methylated peaks of SOX9 mRNA; (D) M$^6$A methylated peaks of CD34 mRNA; (E) M$^6$A methylated peaks of FGFR2 mRNA; (F) M$^6$A methylated peaks of LEF1 mRNA; (H) M$^6$A methylated peaks of TRPV3 mRNA; (I) M$^6$A methylated peaks of WNT2 mRNA; (J) M$^6$A methylated peaks of BMP4 mRNA; (K) M$^6$A methylated peaks of FGF5 mRNA; (L) M$^6$A methylated peaks of EDAR mRNA; (M) M$^6$A methylated peaks of LIPH mRNA; (N) M$^6$A methylated peaks of MSX2 mRNA; (O) M$^6$A methylated peaks of IGF1 mRNA; (P) M$^6$A

methylated peaks of ETS1 mRNA; (Q) $M^6A$ methylated peaks of EGFR mRNA; (R) $M^6A$ methylated peaks of HOXC13 mRNA; (S) $M^6A$ methylated peaks of WNT5A mRNA; (T) $M^6A$ methylated peaks of RUNX2 mRNA; (U) $M^6A$ methylated peaks of WNT10B mRNA.
